# Our Theater of Anonymity

**DOI:** 10.1002/eahr.60027

**Published:** 2025-07-14

**Authors:** Sara Meeder, Megan Doerr

**Affiliations:** ^1^ Assistant vice president of research administration at Maimonides Health; ^2^ Director at Sage Bionetworks

**Keywords:** human research ethics, ethics review boards, big health data research, identifiability, deidentified data

## Abstract

Ethics review boards are increasingly asked to review big health data research proposals using a regulatory framework written prior to the current era of machine learning and artificial intelligence. Traditional consideration of individual identifiability does not account for the growing recognition that almost all data can be reidentified. This leaves the research ethics community performing a “theater of anonymity”: weighing benefit versus risk on the inclusion of participant identifiers alone. The wider research community, including U.S. federal agencies, is pushing for greater transparency and data sharing, stretching the current definition of identifiability to the breaking point. Additionally, shifting attitudes on the balance between privacy and research benefit may make a project more acceptable to individuals and communities than to the ethics boards designed to serve them. Reviewing the human research community's historical and current understanding of identity in terms of research, risk, benefit, and consent may point toward a need for a change in how consent is conceived. Particularly in the context of consent for big health data research, the research ethics community may need to shift its focus from solely considering individuals toward actively considering the interests of the communities most likely to be affected by the research.

Whether we are ready to admit it or not, the research ethics community is in the midst of a privacy arms race. Almost daily, another publication describes a “new” data type or computational approach that dismantles traditional notions of privacy.^1^ Electronic health record data, multi‐omics profiling, and digital data from wearable devices—not to mention other online activities from banking to health care to social media—create unique digital phenotypes that, when combined with publicly available data, can be connected to the source individuals.^2^ Add to this ever‐advancing interrogation approaches in machine learning and artificial intelligence, and research participants may quickly (and inadvertently) be re‐identified.^3^

In response, the research community develops and implements privacy preserving data governance approaches: synthetic data, sandboxing (“walled gardens”), federated learning, and differential privacy (“model to data”)—the list goes on.^4^ Despite their value to data controllers and regulators as privacy stopgaps, each of these data governance approaches comes at a price, limiting the science that can be done with these incredibly valuable data in some important way,^5^ which begs the question, why do we persist in this unwinnable contest?

How has the “theater of anonymity” become so central to research ethics?^6^ And, to that end, can we acknowledge that there may be circumstances where individuals (and communities) may value the benefits of research over (increasingly improbable) promises of privacy?

The idea of identifiability varies across disciplines and regulations. The Health Insurance Portability and Accountability Act of 1996, a federal law that dictates how certain entities can use and disclose protected health information, lists 18 categories of information that are considered to be identifying alone or in combination with other information.^7^ The regulations governing federally funded human subjects research (i.e., the Common Rule), does not parse out specific identifying variables, but instead refers to identifiable private information, which is information or a biospecimen that can readily identify a research participant.^8^ The lack of specificity in the Common Rule can cause difficulties when assessing the potential risks of reidentification of research study data, particularly in light of the growing complexity of the nature of identifiers themselves.^9^

Under the Common Rule, ethics boards currently consider readily identifiable data as data that would quickly render the individual identities of research participants apparent to an investigator—such data as participants’ names or home addresses. When reviewing proposed research, committee members are tasked with determining the level of potential risk associated with the study.^10^ One element of risk assessment is based on the potential for harm due to breach of data, making it important to understand the sensitivity and identifiability of the data or biospecimens. It is interesting to note that, within the context of the Common Rule, the risks of identifiability are considered only in terms of the individual. By contrast, the Common Rule conceptualizes the benefits of research in terms of both individual benefit and group/societal benefit. Although we previously explored the origins of the Common Rule's preclusion against considering group harm, it seems important to think about the shift in federal regulators’ stance regarding identifiability and risk.^11^

To better understand how the research community has arrived at its current conception of identifiability and consent, it helps to look at how regulatory changes have addressed the notion of consent given the shifting mosaic of technical advances and ethical considerations. The Department of Health and Human Services published an Advanced Notice of Proposed Rulemaking (ANPRM) regarding the Common Rule in 2011, citing, as one of the reasons for the proposed revision, that “the system has been criticized as not adequately calibrating the review process to the risk of research.”^12^ The ANPRM referred to the changing research landscape and complaints about over‐regulation of some types of research, putting the proposed revision in terms of better protecting research participants while reducing investigator burden. The method for meeting these dual goals was a puzzling juxtaposition involving the reduction of oversight of research using identifying data with the proposal to increase regulatory oversight for secondary research with previously collected biospecimens.

The proposed Common Rule revision included a requirement for prospective consent for secondary research with biospecimens. In the conversation around the ANPRM and the 2015 Notice of Proposed Rulemaking (NPRM), the recategorization of deidentified biospecimens as being included under the definition of human subjects seems to be a reaction to media coverage due to multiple historical and more recent misappropriations/misuses of biospecimens by the research community.^13^ One highly publicized incident of misappropriation is the story of Henrietta Lacks and the HeLa cell line, which was created from Lacks's biospecimens without her knowledge or consent.^14^ The ramifications of the incredibly profitable “immortal” cell line arising from an incidence of clinical misappropriation without the knowledge of or recompense to Lacks's family played out in the media and courts until 2023, only concluding with the family settling a lawsuit against the technology company responsible for the HeLa line.^15^ There have been many other incidents that have involved the collection and use of biospecimens outside of the purpose conveyed to the populations donating samples, including uses that were contrary to the biospecimen donors’ cultural beliefs and practices,^16^ some or all of which may have influenced decision‐making regarding the proposed rule changes. The ANPRM and NPRM proposed changes to the definition of human subjects to include deidentified biospecimens was suggested to allow greater autonomy to those whose biospecimens were collected clinically (not specifically for a research study).^17^ Use of those clinically collected biospecimens for research purposes under the proposed rule change would have required obtaining consent from the biospecimen “donor.” At the same time, during a town hall meeting on the Common Rule NPRM held in 2015, representatives from the U.S. Office for Human Research Protections (OHRP) repeatedly highlighted that the consent requirement for previously collected, deidentified biospecimens did not apply to data, despite reference to the massive amounts of data involved in internet research and genome sequencing. Menikoff, then the director of the OHRP, characterized the risks of data‐intensive studies as “informational” rather than physical. Informational harms are those that arise due to the exposure of personal information.^18^

The proposed change to the definition of human subject ultimately did not make it into the revised Common Rule due to pushback from the research community, but the reasoning behind the suggested change in definition was particularly interesting when compared to the ANPRM and the NPRM move to lessen regulatory burden for studies involving identifying data. Exempt category 4 was previously only applicable to studies involving identifying clinical data/biospecimens that had been collected in the past. The revised Common Rule now allows for exempt category 4 to be applicable to studies involving clinical data/biospecimens clinically collected in the past or in the future.^19^ Exempt category 4, in its new form, is remarkably similar to expedited category 5, which allows for use of data/biospecimens collected in the past or future. This update has important implications for research oversight: projects falling into the Common Rule's expedited review categories face a higher degree of scrutiny than projects falling into exempt categories. For medical chart reviews and big health data research involving data collected for clinical purposes, IRBs are now shifting their oversight practices from expedited category 5 to exempt category 4. This categorical shift also has implications for informed consent: exempt research does not have a consent requirement.^20^ Exempt category 4 was previously only applicable to studies involving identifying clinical data/biospecimens that had been collected in the past. The revised Common Rule now allows for exempt category 4 to be applicable to studies involving clinical data/biospecimens collected for clinical purposes in the past or in the future. The revised Common Rule also created two new exempt categories, 7 and 8, that involve limited IRB review of studies using identifiable biospecimens/data collected for purposes other than research if broad consent for these biospecimens/data has been previously obtained. The idea of broad consent, where a human subject consents for future use of identifiable biospecimens and data, appears to be a nod to the ANPRM and NPRM plans to require consent for deidentified biospecimens, although whether consenting to unspecified use of personally identifiable data/biospecimens in perpetuity with few restrictions is a win for individual autonomy is debatable.

Returning to Menikoff's statement that the risks of data‐intensive studies are “informational” rather than physical, how might this reframe reidentifiability risks?^21^ Informational harms, in the regulatory context, are directly associated with individual identifiability,

**Research ethics professionals must refocus their efforts, letting go of the increasingly meaningless regulatorily defined deidentification standard—our theater of anonymity—and turning their attention to developing systems that assess the balance point of the relative benefit and risk of anonymity within diverse groups/communities over time**.

and the preclusion for considering group harms in the Common Rule. Ethics review boards are tasked with considering the potential benefits of a proposed study to individuals and society, in comparison to an analysis of potential risks only to the individual or to the participant pool to be enrolled in the proposed study per the Common Rule at 45 C.F.R. 46.111.^22^ Possible scientific merit and societal benefit are envisioned by the human research ethics community from a distance, separated from the “society” we decide may benefit.

For some communities, the “readily identifiable” standard stymies their desire for research, inhibiting exercise of both participant autonomy and the “right to science.”^23^ Many in the genetic and rare disease communities have argued that the benefit of research may be worth the individual and community risks to privacy that open approaches to data sharing entail.^24^ As Clayton and colleagues have observed with regard to genetic privacy, the research ethics community needs to engage with the relevant community from which research participants will be recruited to understand the factors—sociocultural, relational, and otherwise—that influence people's concerns and choices about genetic privacy; the same holds true for other forms of big health data.^25^ They point out that “it is critical to identify the social practices that will make the collection and use of these data more trustworthy for participants.” While there are institutions and groups working on engaging communities in research on various levels, the push for engagement has not been robust or evenly distributed across the research ethics community.^26^

Although there are certainly reasons to be concerned about individual harm from big health data research, there is increasing reason to refocus on concern for informational harms to groups.^27^ The balance between risk and benefit in research vis‐à‐vis ready identifiability and moral good from research is a question for the communities who might benefit from the research themselves, not for the research community to arbitrarily determine.^28^

Past initiatives to give participants greater agency over the use of their personal data and biospecimens relied on individual consent. Part of the reason the NPRM's proposal to require consent for future research use of clinically collected biospecimens failed was because the consent requirement would make biobanks created prior to the Common Rule revision unusable.^29^ The mechanism of individual consent is not typically feasible when dealing with large volumes of biospecimens or data. And, certainly, the goal is not to hinder research, but to encourage meaningful engagement to ensure research use is congruent with individual and community interests. Consent as an individual right and responsibility in big health data research needs to be reconceived in the form of group engagement.

Research ethics professionals must refocus their efforts, letting go of the increasingly meaningless regulatorily defined deidentification standard—our theater of anonymity—and turning their attention to developing systems that assess the balance point of the relative benefit and risk of anonymity within diverse groups/communities over time. Fortunately, there is ample scholarship to help guide this work: from the rare disease community to disability rights, LGBTQ+ activists, and others, evidence for community‐specific “social license” for research abounds.^30^ Social license, a concept born out of community‐industry relations in the 1990's, foregrounds community‐specific social norms and expectations, in addition to legal requirements, as the basis for trust. In the research context, an example of evidence of social license are slogans like the disability community's, “nothing about us without us.”^31^ Moreover, Tribal and Indigenous scholarship and legal precedent, as well as legal conversations of other nations (not Tribal), may also be informative to our understanding of social license in communities who do not benefit from explicit legal recognition.^32^ Given Tribal and Indigenous disillusionment with most important big health data research efforts, we in the research ecosystem—researchers, oversight professionals, funders—disregard community expectations regarding research to our peril.^33^ As Menikoff observed at the October 2015 OHRP town hall meeting regarding proposed changes to the Common Rule, “The goal is [asking]: should we change? Can we change our rules protecting research subject[s] in a way that is befitting the type of research we are doing these days?”^34^
